# Distinct DNA repair mechanisms prevent formaldehyde toxicity during development, reproduction and aging

**DOI:** 10.1093/nar/gkae519

**Published:** 2024-06-19

**Authors:** Matthias Rieckher, Christian Gallrein, Natividad Alquezar-Artieda, Nour Bourached-Silva, Pavana Lakshmi Vaddavalli, Devin Mares, Maria Backhaus, Timon Blindauer, Ksenia Greger, Eva Wiesner, Lucas B Pontel, Björn Schumacher

**Affiliations:** Institute for Genome Stability in Aging and Disease, Medical Faculty, University and University Hospital of Cologne, Joseph-Stelzmann-Str. 26, 50931 Cologne, Germany; Cologne Excellence Cluster for Cellular Stress Responses in Aging-Associated Diseases (CECAD) and Center for Molecular Medicine (CMMC), University of Cologne, Joseph-Stelzmann-Str. 26, 50931 Cologne, Germany; Institute for Genome Stability in Aging and Disease, Medical Faculty, University and University Hospital of Cologne, Joseph-Stelzmann-Str. 26, 50931 Cologne, Germany; Josep Carreras Leukaemia Research Institute (IJC), Ctra de Can Ruti, Camí de les Escoles s/n, 08916 Badalona, Barcelona, Catalonia, Spain; Josep Carreras Leukaemia Research Institute (IJC), Ctra de Can Ruti, Camí de les Escoles s/n, 08916 Badalona, Barcelona, Catalonia, Spain; Institute for Genome Stability in Aging and Disease, Medical Faculty, University and University Hospital of Cologne, Joseph-Stelzmann-Str. 26, 50931 Cologne, Germany; Institute for Genome Stability in Aging and Disease, Medical Faculty, University and University Hospital of Cologne, Joseph-Stelzmann-Str. 26, 50931 Cologne, Germany; Institute for Genome Stability in Aging and Disease, Medical Faculty, University and University Hospital of Cologne, Joseph-Stelzmann-Str. 26, 50931 Cologne, Germany; Institute for Genome Stability in Aging and Disease, Medical Faculty, University and University Hospital of Cologne, Joseph-Stelzmann-Str. 26, 50931 Cologne, Germany; Institute for Genome Stability in Aging and Disease, Medical Faculty, University and University Hospital of Cologne, Joseph-Stelzmann-Str. 26, 50931 Cologne, Germany; Institute for Genome Stability in Aging and Disease, Medical Faculty, University and University Hospital of Cologne, Joseph-Stelzmann-Str. 26, 50931 Cologne, Germany; Josep Carreras Leukaemia Research Institute (IJC), Ctra de Can Ruti, Camí de les Escoles s/n, 08916 Badalona, Barcelona, Catalonia, Spain; Instituto de Investigación en Biomedicina de Buenos Aires (IBioBA), CONICET - Partner Institute of the Max Planck Society, C1425FQD, Buenos Aires, Argentina; Institute for Genome Stability in Aging and Disease, Medical Faculty, University and University Hospital of Cologne, Joseph-Stelzmann-Str. 26, 50931 Cologne, Germany; Cologne Excellence Cluster for Cellular Stress Responses in Aging-Associated Diseases (CECAD) and Center for Molecular Medicine (CMMC), University of Cologne, Joseph-Stelzmann-Str. 26, 50931 Cologne, Germany

## Abstract

Formaldehyde (FA) is a recognized environmental and metabolic toxin implicated in cancer development and aging. Inherited mutations in the FA-detoxifying enzymes *ADH5* and *ALDH2* genes lead to FA overload in the severe multisystem AMeD syndrome. FA accumulation causes genome damage including DNA–protein-, inter- and intra-strand crosslinks and oxidative lesions. However, the influence of distinct DNA repair systems on organismal FA resistance remains elusive. We have here investigated the consequence of a range of DNA repair mutants in a model of endogenous FA overload generated by downregulating the orthologs of human *ADH5* and *ALDH2* in *C. elegans*. We have focused on the distinct components of nucleotide excision repair (NER) during developmental growth, reproduction and aging. Our results reveal three distinct modes of repair of FA-induced DNA damage: Transcription-coupled repair (TCR) operating NER-independently during developmental growth or through NER during adulthood, and, in concert with global-genome (GG-) NER, in the germline and early embryonic development. Additionally, we show that the Cockayne syndrome B (CSB) factor is involved in the resolution of FA-induced DNA–protein crosslinks, and that the antioxidant and FA quencher *N*-acetyl-l-cysteine (NAC) reverses the sensitivity of detoxification and DNA repair defects during development, suggesting a therapeutic intervention to revert FA-pathogenic consequences.

## Graphical Abstract

**Graphical Abstract:**
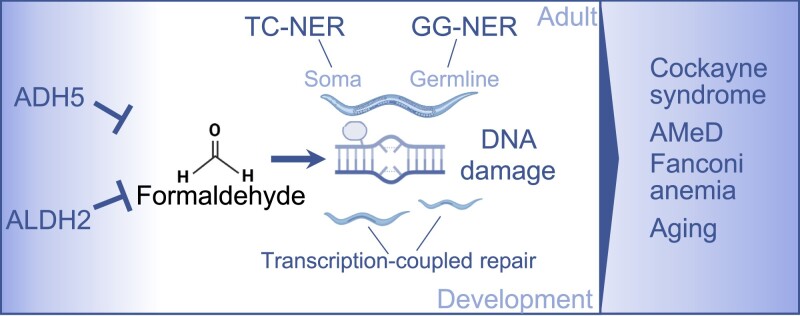

## Introduction

Formaldehyde (FA) is a widely recognized environmental cytotoxin and genotoxin. It is a simple organic compound that can be found in various products, including building materials, household products and vehicle emissions (1). Additionally, FA is generated within cells through several pathways, such as the metabolization of methanol, the demethylation of proteins and nucleic acids, and as a product of the breakdown of the one-carbon metabolism carrier tetrahydrofolate (2,3). FA exhibits the capacity to induce diverse forms of damage to cellular biomolecules (4,5). In the genome, it can form covalent crosslinks between DNA strands, both inter (ICL) and intra-strands (ISL) and between DNA and proteins (DPC) (6). FA might also chemically modify DNA bases, altering their structure and functionality. These DNA lesions could cause replication stress, single-strand breaks (SSB) and double-strand breaks (DSB) requiring multiple DNA repair mechanisms to guarantee genome stability. Failure to repair these lesions could obstruct the normal processes of DNA replication, transcription and repair, resulting in genomic instability and compromised DNA integrity.

Several DNA repair pathways have been implicated in the cellular response to FA-induced genotoxic stress (7,8), including the interstrand-crosslinking (ICL) Fanconi anemia DNA repair pathway; the CSB protein, which initiates transcription-coupled repair (TCR) when RNA polymerase II stalls at a transcription blocking lesion; and the exonuclease 1 (EXO1) (9,10). In animal models, the genetic factors that determine endogenous FA genotoxicity have been investigated upon inactivation of a DNA repair pathway in combination with the enzyme alcohol dehydrogenase 5 (ADH5), which is a central factor in the glutathione (GSH)-dependent detoxification of FA (7). Specifically, in mice, the Fanconi anemia DNA repair pathway factor D2 (Fancd2) and the transcription coupled factor Csb have been shown to genetically interact with Adh5, indicating a central role of these DNA repair factors in counteracting the genotoxicity of cellular FA in animals (9,10).

FA has emerged as a fundamental contributor to human disorders such as Fanconi anemia, Cockayne syndrome, and AMeDS, which are caused by mutations in *FANC* genes, *CSB* or *CSA*, and *ADH5* and *ALDH2*, respectively (11). AMeDS (OMIM #619151) entails a severe bone marrow failure progressing to myelodysplastic syndrome (MDS) and myeloid leukemia (AML) (12). In this condition, inherited inactivating mutations in both alleles of *ADH5* combined with the dominant negative single nucleotide polymorphism r671 in *ALDH2* (c.1510G > A; p.E504K; ALDH2*2), which codes for the mitochondrial enzyme aldehyde dehydrogenase 2, result in systemic FA accumulation driving AMeDS-associated traits. Notably, mice deficient in both *Adh5* and *Aldh2 (Adh5^−/−^Adh2^−/−^)*, or animals carrying the *Aldh2rs671* defective allele in an *Adh5*-deficient background (*Adh5^−/−^Aldh2^−/rs671^*) recapitulated most of the human disease phenotypes, supporting the role of endogenous FA in driving AMeDS (12,13). A prevailing hypothesis suggests that FA-induced damage overwhelms DNA repair mechanisms, yet the extensive array of FA-induced lesions might necessitate safeguarding the genome by multiple DNA repair systems. However, establishing genetic evidence in animals to validate these mechanisms remains experimentally challenging.

Here, we employed the genetically traceable animal model organism *C. elegans* to explore DNA repair components safeguarding the genome against FA-induced damage. *C. elegans* undergoes most cell divisions during embryogenesis and hatches as L1 larvae that are comprised of mostly differentiated somatic cell types that grow in size during the L2, L3 and L4 larval stages until adulthood. Germ cells, in contrast, remain replicative active throughout adulthood. We first systematically determined the contribution of a wide range of DNA repair mechanisms to the survival of FA treatment in the presence and absence of *adh-5*. Based on these results, we further determined tissue and development specific roles of NER components for FA tolerance. CSB-induced NER-independent TCR was required for FA resistance in postmitotic somatic tissues during developmental growth, while CSB-induced NER-dependent TCR was required in adulthood. In contrast, GG-NER was essential in germ cell and embryonic development. We further identified the ortholog of human ALDH2 (*alh-1*) and developed a nematode model of AMeDS, ascertaining the roles of TCR and NER in this model. Lastly, we determined that N-acetyl cysteine (NAC) supplement could effectively restore FA resistance suggesting a potential intervention strategy for AMeDS.

## Materials and methods

### *C. elegans* maintenance

*Caenorhabditis elegans* were maintained on *Escherichia coli* strain (OP50) seeded NGM plates at 20°C (29). The strains BJS885, BJS938, BJS939 and BJS940 were produced via standard genetic crossing in the Schumacher lab. Strain BJS886 was created through CRISPR/Cas9 as published previously (4). The complete list of strains is included in the Table 1.

### Human cell lines

Cell lines used in this study were obtained from Coriell repository. Cells were tested free for mycoplasma infection and no further genetic validation was performed. Transformed fibroblasts were grown in Dulbecco's modified Eagle's medium (DMEM) (#10-013-CV, Corning), 10% FBS (Gibco) containing antibiotic/antimycotic mix (#L0010, Biowest). EBV-immortalized lymphoblastic cells were grown in Roswell Park Memorial Institute (RPMI) #61870044 (Gibco),10% FBS containing the antibiotic/antimycotic mix.

**Table:** 

Cell line ID	Information
GM00637	Apparently healthy transformed fibroblasts
GM01712	Cockayne syndrome, type b; CSB, EBV-lymphoblastic cells
GM04312	Xeroderma pigmentosum, complementation group A, transformed fibroblasts
GM06914	Fanconi anemia, complementation group A, transformed fibroblasts
GM09942	Xeroderma pigmentosum, complementation group C, EBV-lymphoblastic cells
GM12496	Cockayne syndrome, type A; EBV-lymphoblastic cells
GM13022	Fanconi anemia, complementation group A, EBV-lymphoblastic cells
GM13295	Xeroderma pigmentosum, complementation group A, EBV-lymphoblastic cells
GM15983	Xeroderma pigmentosum, complementation group C, transformed fibroblasts
GM16094	Cockayne syndrome, type A; CSA transformed fibroblasts
GM16095	Cockayne syndrome, type B; CSB transformed fibroblasts
GM26200	Apparently healthy individual; EBV-lymphoblastic cells

### FA-toxicity assays

For a standard FA-toxicity assay as in Figure 2, worm populations were grown up to gravid adult stage on nematode growth medium (NGM) plates and synchronized via sodium-hypochlorite treatment (5 M NaOH and sodium hypochlorite in a 1:1 ratio), followed by three washes with M9 (3 g KH_2_PO_4_, 6 g Na_2_HPO_4_, 5 g NaCl, in 1 l H_2_O; autoclaved and added 1 ml of 1 M MgSO_4_). The eggs were grown in M9 for 16–18 h on a roller shaker overnight in the dark at room temperature. Synchronized L1 larvae were seeded on OP50-seeded NGM plates with or without FA (methanol-free 16% FA, Thermo Scientific) in the indicated concentrations and scored for developmental timing and survival after 48 and 72 h at a stereomicroscope.

For the egg laying assays in Figure 3, animals were grown into a population of gravid adults and synchronized via sodium-hypochlorite treatment as indicated above. Synchronized animals were then grown on OP50-seeded NGM plates for exactly 72 h to reach adulthood. Three animals were transferred to fresh OP50-seeded NGM plates with or without FA in the indicated concentrations and allowed to lay eggs for 3 h. Eggs were counted thereafter, and hatching was determined 24 h later.

**Figure 1.**
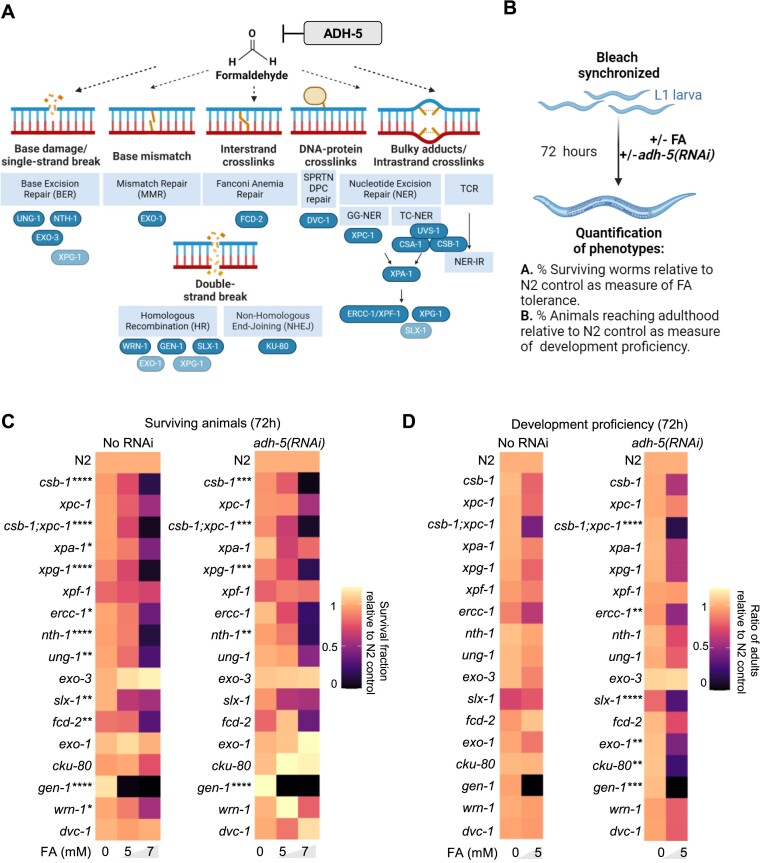
DNA repair determinants of formaldehyde tolerance in *C*.*elegans*. (**A**) Cartoon showing the putative lesions caused on DNA by formaldehyde (FA). In blue the factors inactivated in this work. Light blue shows some factors that might participate in multiple DNA repair pathways. (**B**) Scheme of the protocol applied to address FA tolerance and development in the data shown in C and D (see Materials and methods for details). (**C**) Heatmap summarizing survival data for *C. elegans* mutants in DNA repair and upon downregulation of *adh-5* via RNAi. (**D**) Heatmap summarizing animals reaching adulthood as measure of development proficiency for *C. elegans* mutants in DNA repair and upon silencing of ADH-5. The heatmaps contain the mean value of three independent biological replicates for each condition. The significance was determined via a two-way ANOVA and the Dunnett's multiple comparisons test, for which counts *P < 0.05, **P < 0.01, ***P < 0.001, ****P < 0.0001. The full statistical analysis for (C) and (D) is available as supplementary tables. Cartoons were created with BioRender.com.

For the FA-toxicity assays combined with RNAi-mediated downregulation of *adh-5* in Figure 1, we used HT115 bacteria expressing dsRNA against *adh-5(RNAi)*, as published previously (4). The bacterial strains were propagated overnight in LB containing Ampicillin (0.1 mg/ml) and Tetracycline (0.0125 mg/ml) for selection. The overnight culture was diluted 1:100 into 100 mL LB liquid with Ampicillin and grown for 6 h, before Isopropyl β-d-1-thiogalactopyranoside (IPTG, 1 mM) for dsRNA-induction was added and grown for another hour. The resulting culture was concentrated 10 times by centrifugation and seeded on NGM plates containing IPTG (1 mM), Ampicillin (0.1 mg/ml) and with or without FA in the indicated concentrations. The bacteria on the plates were allowed to grow overnight at room temperature before the worms were added, and developmental timing and survival were scored after 48 and 72 h later.

For experiments downregulating *alh-1* in Figure 5, we isolated *alh-1(RNAi)* from the Ahringer genome-wide RNAi library (Fwd primer sequence GGGAGTCTGCGGACAGATTA, Rev primer sequence CAGGGAAAGCGAAAATTCAA, Source Bioscience location III-2N01) and prepared the RNAi plates as indicated above (30). We exposed the P0 generation to RNAi from L1 stage for 72 h before transferring the adults to fresh RNAi plates with or without FA in the indicated concentrations for 24 h. The animals were transferred to fresh RNAi plates and allowed to lay eggs for 3 h. Then, the adults were removed, and the eggs were counted, and hatching was scored after 24 h. After 48 h we determined the developmental stages and re-examined the plates for dead embryos. For GSH-precursor experiments in Figure 6, we added NAC (LINARIS Biologische Produkte GmbH, Germany) into the NGM agar at a 10 mM concentration.

To determine cell viability, we used a resazurin-based assay. Briefly, cells were seeded at 3000 cells/well in 96-well plates and exposed to the concentrations of FA depicted in the corresponding figures (prepared from Formaldehyde Solution 16%, #28906, Thermofisher). After 3 (for fibroblast) or 6 (for EBV-lymphoblastic cells) days resazurin (#418900050, Thermofisher) was added at a final concentration of 44 μM for 4 h and fluorescence quantified. Data is presented as % of the untreated samples unless otherwise stated. In the indicated plots *N*-acetyl-l-cysteine (#A7250, Sigma-Aldrich) was used at 500 μM.

### Comet assay

For the analysis of DNA damage burden, approx. 250 day-1-old adult nematodes were subjected to the comet assay 24 h after on-plate FA exposure. UVB-treated animals (24 h post-treatment) served as a positive control (20). Nematodes were washed 10 times for three min with M9 to remove residual bacteria. Afterwards, the cuticle was permeabilized by adding 200 μl TSD buffer (0.5% Triton X-100, 20 mM HEPES buffer (pH 8.0), 0.25% SDS, 200 mM DTT and 3% sucrose) for four min. Permeabilization was stopped by adding 800 μl egg buffer (25 mM HEPES (pH 7.3), 118 mM NaCl, 48 mM KCl, 2 mM CaCl_2_, 2 mM MgCl_2_). Animals were washed five additional times with egg buffer and then dissociated to single cells by adding 150 μl pronase (15 mg/ml in egg buffer) and mechanical disruption (pipetting up and down 160 times with a P1000 pipette within 20 min incubation at RT). The digestion was stopped by adding 1 ml ice cold FCS (Sigma-Aldrich) and cells were pelleted in a centrifuge (rotating at 9600×g at 4°C for 5 min). Cells were washed with PBS, pelleted by centrifugation, and finally resuspended in 200 μl PBS. Cells were allowed to settle on ice for 30 min and passed through a 40 μm cell strainer. 50 μl of cells were mixed with 150 μl low melting agarose (0.65% in H_2_O, temperature adjusted to 42°C in waterbath); 75 μl of the cell-agarose mix was spread on a slide (precoated with 1% agarose, air-dried). Slides were placed in a Coplin jar and embedded cells were lysed in lysis buffer (2.5 M NaCl, 100 mM EDTA, 1% (v/v) Triton X-100, 0.3% (w/v) Lauroyl sarcosin, 10 mM Tris-base, 250 mM NaOH, adjust to pH 10.0) at 4°C overnight. The next day, slides were washed three times with H_2_O at 4°C for 5 min and subjected to unwinding buffer (0.3 M NaOH, 1 mM EDTA) at 4°C for 60 min. Afterwards, slides were transferred to an electrophoresis chamber (filled with unwinding buffer) and run at 22 V (≈200 mA) for 24 min. After running, slides were transferred to neutralization buffer (0.4 M Tris–HCl (pH 7.5)) for 10 min, washed three times with H_2_O for 5 min, and finally fixed in methanol for 10 min. The fixed slides were air-dried. 25 μl ethidium bromide (EtBr) was added to the slides and covered with a coverslip. Imaging was performed directly after adding EtBr using an Axio Imager.M2 (Carl Zeiss). 14-bit images were acquired at 100-fold magnification using an RFP filter set. 30–50 comets per condition were analyzed using Fiji, applying the circle tool to measure ‘Area’ and ‘IntegratedDensity’ of the comet head and the total comet. Additionally, the ‘Length’ of the comet tail was measured. Measured values were corrected for the median background of their respective image. Tail moment was calculated as (tail length) × (% of DNA in comet tail)). As a positive control, 250 day-1-adult, wild type worms were placed on a non-seeded plate and irradiated with 300 mJ/cm^2^ UVB (broad-band spectrum) directly before being subjected to the initial washing steps of the comet assay.

### DNA-protein crosslink assay

DPCs caused by FA were isolated using the ARK method (31). Briefly, 300 000 cells of GM00637 and GM16095 were seeded per well in 6-well-plates. After 48 h, the culture media was replaced with fresh media containing 0, 200 and 400 μM of FA. Once the treatment reached 4 h, half of the samples were kept in culture with media without FA to recover for 24 h, and the rest were pelleted. After the collection of the pellets at the end of the assay, samples were lysed with a guanidine thiocyanate-based buffer, and DNA was isolated with a 50% ethanol precipitation method. DPCs and free DNA were separated by dissolving the pellet with a solution containing 1% SDS, and precipitating DPCs using a K–Cl buffer. DPC precipitate was digested with 0.2 mg/ml proteinase K (#BP1700-100, Thermofisher) to release DNA from covalently bound proteins. Following the procedure, DNA quantification of free DNA and DNA isolated from DPC precipitate was performed using Qubit dsDNA Quantification Assay Kit (#Q32851) as manufacturer's instructions. Finally, the DPC coefficient was calculated as follows: DNA isolated from DPC/total DNA.

### Protein Immunoblotting

Cell lines were lysed using RIPA buffer (50 mM Tris–HCl pH7.4; 150 mM NaCl; 1% sodium deoxycholate; 0.10% SDS; 1% Triton-X100; 0.5 mM EDTA) supplemented with protease- (Roche #04693116001), and phosphatase inhibitors (Roche #04693116001) and sonicated. 60 μg of protein lysate was loaded for each sample onto a 15% acrylamide gel in a 1.0 mm Mini Protein Gel (Invitrogen), and subsequently transferred to a 0.1 μm nitrocellulose membrane. The running conditions were set at 80 V for the first 15 min, followed by 100 V for 1 h and 45 min. Transfer conditions were set at 100 V for 1 h. The membrane was blocked in 5% BSA in TBS–Tween for 1 h at room temperature. Blots were then incubated on a shaker at 4°C in primary antibodies diluted in 5% BSA in TBS–Tween. Following primary antibody incubation, blots were incubated with fluorescent secondary rabbit or mouse antibodies in 5% BSA in TBS-Tween for 1 hour at room temperature. Immunoblots were washed three times for 5 min each after each incubation. Band intensities were imaged using the Odyssey CLx Imaging System (LI-COR) and subsequently quantified by densitometry using ImageJ software. Finally, the data was plotted using GraphPad. Antibodies used: H2AX 1:1000 ABnova MAB-12332; γH2AX 1:500 Cell Signaling Technology 2577S; TIF1β (KAP1) 1:1000 Santa Cruz Biotechnology Sc-136102; pKAP1 (Ser824) 1:1000 Cell Signaling Technology 4127S; CHK1 1:1000 Santa Cruz Biotechnology Sc-8408; pCHK1 (Ser354) 1:500 Cell Signaling Technology 2348S; IRDye800CW Donkey anti-Rabbit 1:5000 LI-COR 926/32213 IRDye680RD; Goat anti-Mouse 1:5000 LI-COR 926/68070.

### *gst-4*::GFP expression quantification

The strain CL2166 (dv*Is19*[(pAF15)*gst-4*p::GFP::NLS]) was used for oxidative stress signaling induction evaluation (32). The animals were grown on plates with HT115 bacteria expressing control RNAi (empty vector) or RNAi against *adh-5* or *alh-1* for two generations following established RNAi protocols (33). To achieve an equal exposure to RNAi, we measured the OD_600_ of the O/D culture and adjusted equal concentrations to a 50:50 mix. Animals were imaged at a Zeiss AxioImager.M2 with a 5× lens right after a 5-h treatment, and after a 16-h recovery phase.

### Lifespan assay

For lifespan assays, animals were synchronized via sodium-hypochlorite and grown into the larval stage L4 on OP50-seeded NGM plates, before they were transferred to the lifespan assay plates, which contained 50 μM 5-fluor-2′-desoxyuridin (FuDr) to suppress offspring production. Animals were grown with or without 5 mM FA and remained on the same plate until death, while the survival was scored every 2 days. Worms that crawled off the plate or dried out at the edge of plates were censored. The population size was 100–150 worms per strain and condition.

### Phylogenetic analysis

Phylogenetic analysis was performed using Phylogeny.fr suite (34). First, *C. elegans* genes coding for ALDH2-like proteins we retrieved by performing a BlastP restricted to *C. elegans* using human ALDH2 protein as query. The list of proteins was then uploaded into Phylogeny.fr and plotted as phylogram depicting the branch support values.

### Statistical analysis

Statistical significance was assessed via the GraphPad Software. To assess differences in grouped data sets for survival, developmental delay and egg-laying/hatching we used the two-way ANOVA with Tukey's multiple comparisons test unless otherwise stated in the figure legend. For lifespan assays we applied the log-rank (Mantel–Cox) test and the Gehan–Breslow–Wilcoxon test. To test for significant differences in comet assays, one-way ANOVA with Tukey's post hoc test was employed.

### Data availability

All the data reported in this work can be found in the text and supplementary files. Reagents are available from L.B.P. and B.S. upon request.

## Results

### Multiple DNA repair pathways confer FA tolerance in *C. elegans*

To dissect how the multiple DNA repair mechanisms conserved between human and *C. elegans* protect the genome from FA in an animal model, we applied chronic FA-treatment during animal development and measured survival. Among the surviving animals, we scored those that reach adulthood as a measure of development proficiency. We performed this approach in wildtype (N2) animals and in several DNA repair-deficient mutants, including components of TCR and GG-NER, Fanconi anemia, base excision repair (BER), mismatch repair (MMR), non-homologous end joining (NHEJ) and homologous recombination repair (HR) (Figure 1A–C). To exacerbate the effect of FA, we silenced *adh-5* (*adh-5(RNAi)*) in each of the mutants in the DNA repair pathways depicted in Figure 1A. Our analysis revealed that the survival of several mutants for NER (*csb-1*; *xpa-1*; *xpg-1*; *ercc-1*) and of a member of the Fanconi anemia pathway (*fcd-2*), is significantly reduced compared to wild type animals (Figure 1C), which is in line with previous studies that demonstrated an involvement of these pathways in the removal of FA-induced DNA lesions (6,8). We did not detect a significant reduction in survival in animals carrying a mutation for the gene coding the *C. elegans* ortholog of the DPC protease SPRTN (*dvc-1(ok260)*) (14).

Animals carrying mutations in *nth-1* or *ung-1*, which code for components of the BER, were also highly sensitive to FA, which might be a consequence of the property of FA to induce oxidative stress (4). *gen-1* also appeared as a crucial determinant of FA tolerance. The downregulation of *adh-5* did not significantly increase FA toxicity in most of the DNA repair mutants but in contrast appeared to increase survival of *exo-1* and *cku-80* mutants (Figure 1C). However, when analyzing the animals that reach adulthood as a measure of development success, downregulating *adh-5* exacerbated the developmental failure of most of the DNA repair mutants (Figure 1D). The strain *xpc-1;csb-1;adh-5(RNAi)* was one of the most compromised in this development assay indicating NER plays a fundamental role for protecting the larvae during development to adulthood. In this assay, we also observed a contribution of the factors SLX-1, EXO-1, GEN-1 and of the NHEJ component KU-80, suggesting a complex systemic protection of the genome against FA during development and a crucial role of ADH-5 dependent FA detoxification.

### TCR-deficiency is a determinant of somatic FA-sensitivity in *C. elegans* and human cells.

**Figure 2.**
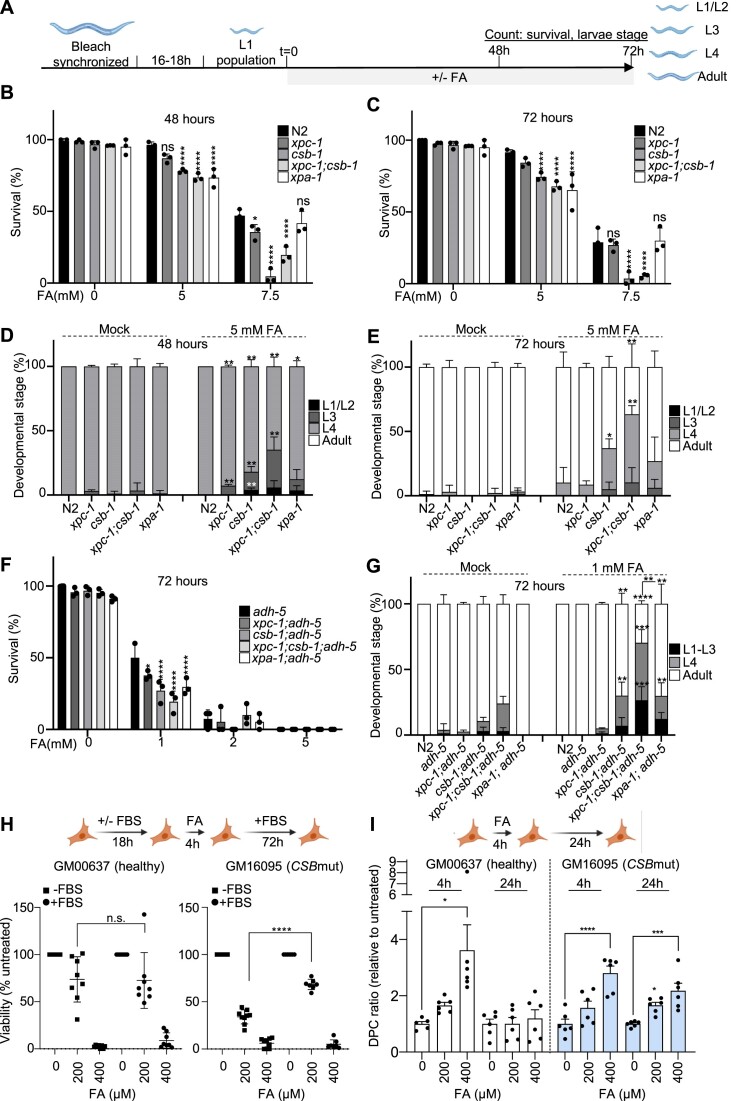
NER-deficient animals show somatic formaldehyde sensitivity, which is further enhanced by loss of ADH-5. (**A**) Experimental scheme (see Materials and methods for details). (**B**) Survival of NER mutants upon formaldehyde (FA) after 48 h and (**C**) after 72 h on plate. (**D**) Developmental timing of NER mutants upon FA after 48h on plate and (**E**) after 72 h on plate. (**F**) Survival of NER mutants in an *adh-5* mutant background upon FA after 72h on plate (for N2 control the survival at 5 mM is 91.5 ± 1.9% as shown in Figure 2C). (**G**) Developmental timing of NER mutants in an *adh-5* mutant background upon FA after 72 h on plate. Each condition was performed in three technical replicates, and the significance was determined via two-way ANOVA for survival, followed by Tukey's multiple comparison test, for which is defined *P < 0.05, **P< 0.01, ***P < 0.001, ****P < 0.0001. (**H**) Top, scheme depicting the experimental set up; bottom viability determined 72 h after the 4 h-FA pulse (*n* = 6, ****P< 0.0001 for a two-tailed *t*-test between the groups indicated in the figure). (**I**) DNA–protein crosslink (DPC) accumulation after a 4 h-FA pulse determined 24 h upon recovery for GM00637 (healthy fibroblasts, left); and for GM16095 (*CSBmut* fibroblasts, right) (*n* = 6, mean ± SEM). One-way ANOVA corrected with Dunnett test for multiple comparison (*P= 0.0109, ****P< 0.0001; *P= 0.0242, ***P= 0.0003). ANOVA tables for panels B, C, D, E and G can be found as supplementary tables. Cartoons were created with BioRender.com.

The significant role of NER components in the protection against FA in conditions of downregulation of systemic catabolism via *adh-5(RNAi*) suggested this pathway might limit FA genotoxicity in animals. Thus, we deepened our analysis on the sensitivity of *C. elegans* mutants for TCR (*csb-1*), which is known to act in the removal of bulky lesions in somatic tissues, and for GG-NER (*xpc-1*), which acts predominantly in the germline (15,16). The distinct roles of TCR and GG-NER are likely due to the reliance of terminally differentiated somatic cells on actively transcribed regions of the genome, while germ cells need to survey their entire genome to ensure inheritance of stable genomes. We also evaluated the double-mutant *xpc-1;csb-1*, as well as *xpa-1*, which acts downstream and coordinates the assembly of the pre-incision complex to remove bulky DNA lesions (17). First, we evaluated the tolerance of *xpc-1*, *csb-1*, *xpc-1*;*csb-1* and *xpa-1* mutants to FA at 48 and 72 h of chronic FA exposure (Figure 2A–C). *csb-1* and *xpc-1;csb-1* mutants were robustly sensitive to FA in these settings (Figure 2C), while *xpc-1* was not sensitive. Interestingly, the developmental timing upon chronic FA-exposure was significantly delayed for *csb-1*, *xpc-1;csb-1*, but not *xpc-1*, while we detected only a mild developmental delay in *xpa-1* mutants (Figure 2D and E).

We next interrogated whether combined loss of DNA repair capacity and cytoplasmic aldehyde-detoxification could lead to a further increase in FA toxicity. We used *adh-5(RNAi)* to sensitize *C. elegans* DNA repair deficient mutants to FA-toxicity. The a*dh5(RNAi)* in WT background showed normal development that is significantly affected by FA exposure, and a significant survival reduction at the highest FA concentration (Supplementary Figure S1A–C). The downregulation of ADH-5 in NER mutants revealed a significant developmental delay for *csb-1* and *xpc-1;csb-1*, while *xpc-1* and *xpa-1* mutants were not additionally affected (Supplementary Figure S1D and S1E). To further validate the role of *ADH-5* and DNA repair in *C. elegans* survival, we crossed *xpc-1*, *csb-1*, *xpc-1;csb-1* and *xpa-1* mutants into the highly FA-sensitive *adh-5* mutant background and evaluated *C. elegans* survival and development in these settings. We found that all NER factors tested contribute to limit the toxicity of FA in condition of chronic exposure at 1 mM (Figure 2F). Curiously, in none of the assays, loss of *xpa-1* resembled the sensitivity of *xpc-1;csb-1* double-mutants, as it is observed in response to UVB-induced DNA damage or ICL-inducing agents (13,17), suggesting a role of CSB-1 in the prevention of FA genotoxicity in somatic tissues that might by independent of its function within the NER. Accordingly, *csb-1* showed a further developmental defect compared to the canonical TC-NER mutants *csa-1* and *uvs-1* (Supplementary Figure S1F, G), and the developmental timing was significantly delayed in *xpc-1;csb-1;adh-5* triple mutant compared to *xpa-1;adh-5* double mutant, further suggesting CSB-1 might play a role in FA tolerance in an NER-independent mechanism (Figure 2G).

Next, we performed assays to determine whether FA could induce DNA DSBs and found a significant accumulation of DNA damage upon 5 and 7.5 mM FA, which was not further enhanced in mutants lacking ADH-5, confirming previous results obtained in human cells ((4), Supplementary Figure S2A).

To further evaluate the genotoxicity of FA in dividing and non-dividing cells, we employed human fibroblasts from healthy and Cockayne syndrome donors and determined whether FA can be differentially affecting non-cycling cells synchronized by serum starvation compared to dividing cells (Figure 2H, top scheme). We found that an acute FA pulse is equally toxic for healthy dividing and non-dividing fibroblasts. In contrast, *CSB*-deficient fibroblasts were significantly more sensitive to FA when they are not dividing (Figure 2H), suggesting CSB might be involved in the resolution of transcriptional conflicts as previously proposed (10). A putative source of transcriptional stress might be the accumulation of DPC, thus we set up to determine DPC formation in healthy and CSB-mutant fibroblasts exposing them to a 4 h acute pulse of FA, and leaving them to recover for 24 h. We found that *CSB*-mutant fibroblasts cannot significantly reduce the levels of DPC compared to healthy fibroblasts (Figure 2I), indicating CSB is involved in the resolution of DPC caused by FA. We also evaluated the DNA damage markers γ-H2AX, pKAP1(Ser824), and pCHK1(Ser354) in dividing and non-dividing fibroblasts. These data showed that a 4 h pulse of 400 μM FA induces DNA damage markers both in cycling and non-dividing cells, supporting that FA can cause multiple types of DNA lesions (Supplementary Figure S2C, S2D).

In sum, these results indicate that FA causes somatic defects, which are in part limited by a CSB-1 mediated TCR that is only partially dependent on NER. These data also suggest that CSB-1 (CSB) predominantly induces NER-independent repair of FA-induced DNA lesions in terminally differentiated somatic tissues, showing that one of such lesions are DPCs.

### Loss of GG-NER or XPA-1 exacerbates FA-induced fecundity defects in *adh-5* mutants

**Figure 3.**
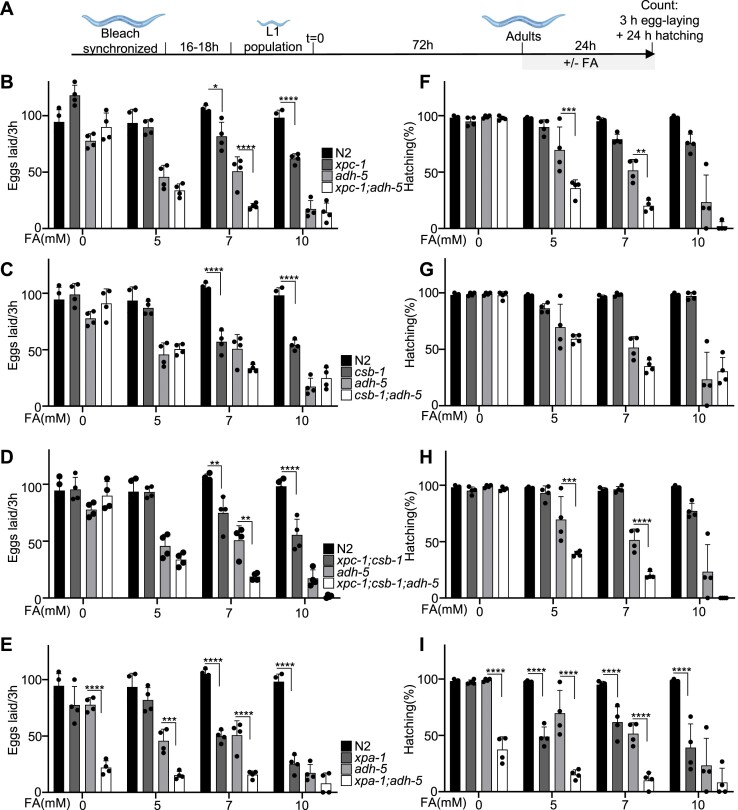
Fecundity is reduced in NER mutants upon formaldehyde (FA) toxicity, which is further enhanced upon loss of ADH-5. (**A**) Experimental scheme (see Materials and Methods for details). Panels (B–E) show the number of eggs produced by three animals within 3 h for strains carrying mutations for (**B**) *xpc-1*, (**C**) *csb-1*, (**D**) *xpc-1*;*csb-1* and (**E**) *xpa-1*. Panels (F–I) display the hatching rate as counted 24 h post-egg-laying for (**F**) *xpc-1*, (**G**) *csb-1*, (**H**) *xpc-1*;*csb-1* and (**I**) *xpa-1*. The graphs summarize the data of four technical replicates, and the significance was determined via the two-way ANOVA, followed by the Tukey's multiple comparison test, for which counts *P< 0.05, **P< 0.01, ***P< 0.001, ****P< 0.0001. ANOVA tables for panels B–I can be found as supplementary tables. Cartoons were created with BioRender.com

FA is a potent environmental toxin that has been directly linked to infertility, reduced offspring and embryonic lethality in humans and mice (7,18,19). To explore the genetic determinants of protection against FA in germ cells and embryos, we exposed adult worms and followed the egg-laying activity and subsequent egg hatching rates upon FA-induced toxicity in *xpc-1*, *csb-1*, *xpc-1;csb-1*, and *xpa-1* mutants in a *adh-5* proficient and deficient backgrounds (Figure 3A). Mutants for *adh-5* showed a reduced number of laid eggs in presence of FA, which was further enhanced upon the loss of *xpc-1* or *xpc-1;csb-1*, but not on *csb-1* alone (Figure 3B–D). Interestingly, *xpa-1;adh-5* double-mutants displayed reduced egg-laying without FA-exposure (Figure 3E), suggesting that NER and ADH-5 are required to limit the toxicity of endogenous FA in the germline.

A normal hatching rate is indicative of a healthy germline. Conversely, reduced hatching has been linked to DNA damage (20). FA caused significantly reduced hatching in animals lacking ADH-5, which was further increased upon loss of XPC-1 (Figure 3F). In contrast, hatching was not affected in *csb-1* mutants even upon loss of ADH-5 (Figure 3G). In line with the survival results, the triple mutant *adh-5;xpc-1;csb-1* presented a striking hatching defect in presence of FA (Figure 3H). Furthermore, loss of XPA-1 led to a reduced hatching rate under control conditions, which worsened upon exposure to FA (Figure 3I). These results show that FA can cause reduced fertility in *C. elegans*, and that detoxification via ADH-5 and genome protection via the GG-NER pathway including XPA-1 have a central role in protecting the germline from FA-induced cytotoxicity. In sum, the removal of FA-induced lesions throughout the genome by GG-NER is required for the inheritance of stable genomes ensuring embryonic health.

### Loss of TCR or XPA-1 further reduces lifespan of adult animals upon FA in *adh-5* mutants.

**Figure 4.**
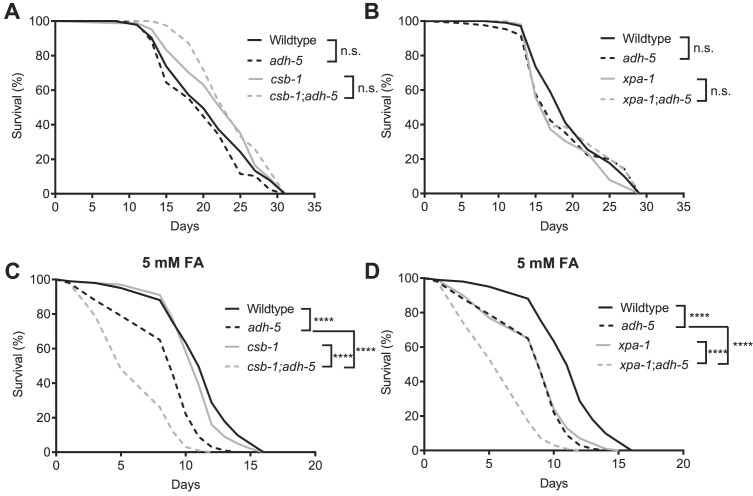
Loss of ADH-5 does not shorten the lifespan of animals deficient for *xpa-1* and *csb-1*, while formaldehyde-sensitivity is increased. (**A**) and (**B**) show full lifespan experiments of wildtype and *adh-5* mutants, while (A) includes *csb-1* and *csb-1*;*adh-5* mutants, and (B) includes *xpa-1* and *xpa-1*;*adh-5* deficient animals. In (**C**) and (**D**), animals were exposed to 5 mM formaldehyde (FA) from the L4 stage and lifespan was determined for (C) animals deficient in *csb-1* and *csb-1*;*adh-5*, and (D) mutants for *xpa-1* and *xpa-1*;*adh-5*. The significance between conditions was determined via the log-rank (Mantel-Cox) test, for which is defined ****P < 0.0001.

Simultaneous loss of Csb and Adh5 leads to reduced survival in *csb^m/m^; adh5^−/−^* double mutant mice (21). To address the effect of FA exposure during adulthood, we determined its effect on lifespan. In *C. elegans*, single loss of ADH-5 or CSB-1, or even simultaneous loss of ADH-5 and CSB-1 did not significantly affect lifespan (Figure 4A). We extended our analysis to *xpa-1;adh-5* mutants and did not detect a significant change in lifespan here either (Figure 4B). FA-exposure significantly reduced the lifespan of *adh-5* mutants (Figure 4C). In addition, loss of either CSB-1 or XPA-1 rendered animals susceptible to FA, which was further enhanced by the loss of ADH-5 (Figure 4C and D), overall indicating that NER-dependent TCR ensures healthy aging in conditions of FA overload.

### Loss of two FA-detoxification systems causes developmental arrest and fecundity defects.

**Figure 5.**
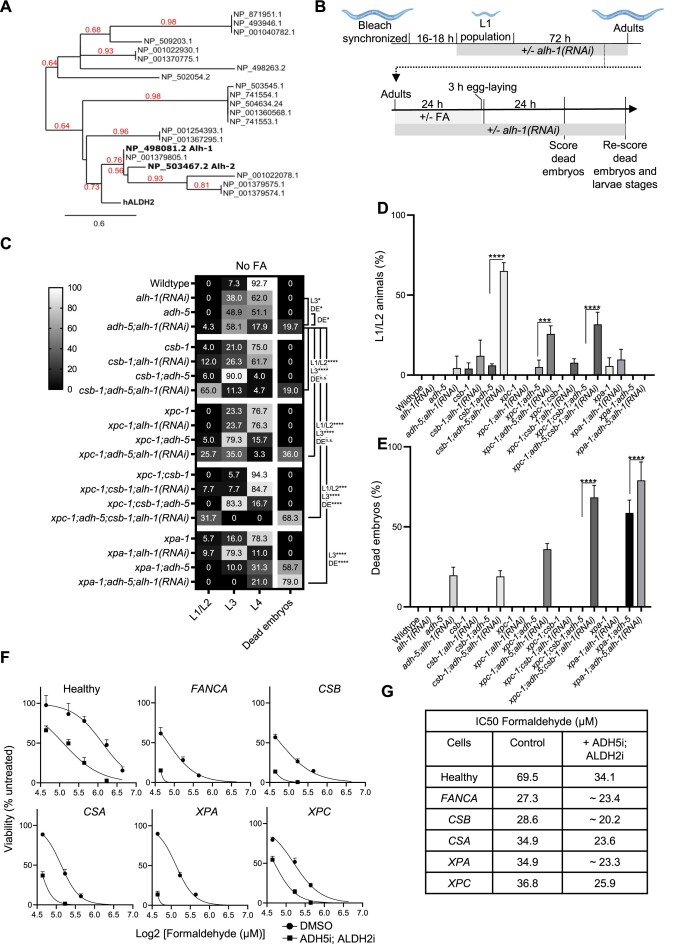
Simultaneous loss of redundant formaldehyde-detoxification pathways causes embryonic lethality and developmental delay, which is enhanced in NER-deficient animals. (**A**) Phylogenetic tree of *C. elegans* proteins showing similarity to human ALDH2. Numbers in red branch support values. (**B**) Experimental scheme (see Materials and methods for details). Cartoons were created with BioRender.com. (**C**) Heatmap combining data of development and embryonic lethality of animals exposed to *alh-1(RNAi)*. The displayed values are the mean percentages of events counted on the plates (stages and dead embryos) after 48 h of development for three technical replicates. The significance was determined via the two-way ANOVA, followed by the Tukey's multiple comparison test, for which counts *P < 0.05, **P < 0.01, ***P < 0.001, ****P< 0.0001. A full statistical analysis between all the groups is available in the supplementary tables. (**D**) Percentages of L1/L2 stage animals for the various mutant backgrounds with *alh-1(RNAi)*. (**E**) Percentages of dead embryos counted on plates. The significance in D and E was determined using a one-way ANOVA followed by the Tukey's multiple comparison test reporting adjusted *p* values for each comparison. (**F**) Viability plots for Epstein-Barr virus (EBV) immortalized lymphoblastic cells from patients with mutations in the genes depicted in the scheme growth. Cells were exposed to formaldehyde (FA) in presence/absence of a cocktail containing the ADH5 inhibitor N6022 (10 μM) and the ALDH2 inhibitor Disulfiram (0.1 μM) (*n* = 3). (**G**) Table showing the IC50 for FA calculated from the data in F using GraphPad.

Systemic FA overload because of inherited mutations in the FA-detoxifying enzyme ADH5 and dominant negative mutations in the mitochondrial aldehyde dehydrogenase 2 (ALDH2) can drive the human condition AMeDS (12). This condition can be recapitulated in mice, still the high penetrance of the postnatal lethality of *Adh5^−/−^ Aldh2^−/−^* mice challenges experimental work with mouse AMeDS models (13). Thus, we set out to develop a *C. elegans* model of human AMeDS. First, we performed a phylogenetic analysis identifying two putative orthologs of human ALDH2 (*alh-2* and *alh-1*) (Figure 5A). Interestingly, only ALH-1 presented the two conserved cysteines in the active site (Supplementary Figure S3A). Furthermore, when combining *adh-5* mutants with RNAi against *alh-1* or *alh-2* we detected that only *alh-1* was able to genetically interact with *adh-5* indicating ALH-1 is the ortholog of human ALDH2 in *C. elegans* (Supplementary Figure S3B). Then, we designed an experiment to evaluate the contribution of ALH-1 and ADH-5 to *C. elegans* development and survival (Figure 5B). First, animals were reared a full generation on *alh-1(RNAi)* starting from L1, which did not cause any detrimental defects in the tested mutants. The following generation showed significantly delayed development on *alh-1(RNAi)*, and, when combined with the loss of ADH-5, we additionally detected a significantly increased embryonic lethality (Figure 5C), overall supporting FA detoxification by ADH-5 and ALH-1 is a conserved mechanism with impact in *C. elegans* development. Together, these data indicate that *C. elegans* could be used as a tractable model for studying some aspects of the human condition AMeDS.

We next explored whether NER plays any role in protecting the genome in animals with almost complete loss of FA catabolism (*adh-5*;*alh-1(RNAi)*). In this background, inactivation of *csb-1* caused a significant developmental delay with 65% of animals arresting at the L1/L2 stage compared to 4.3% for the *adh-5*;*alh-1(RNAi)* control. Loss of XPC-1 (*xpc-1; adh-5; alh-1(RNAi)*) presented with an intermediate phenotype with a 25.7% of animals at L1/L2 stage (Figure 5C,D). Loss of XPA-1 further increased embryonic lethality in an *adh-5*;*alh-1(RNAi)* deficient background (79% dead embryos). The combination of *xpc-1* and *csb-1* mutations in *adh-5;alh-1(RNAi)* animals also showed a striking increase in dead embryos of 68%, and full larval arrest of the hatchlings in the earliest developmental stages (Figure 5D and E). Interestingly, loss of XPA-1 in the aldehyde-detoxification deficient genetic background caused strong embryonic lethality, but offspring developed unperturbed (Figure 5E). Treatment with FA caused a concentration-dependent increase in developmental delay across the different genetic backgrounds, and complete embryonic lethality in animals lacking both ADH-5 and ALH-1 (Supplementary Figure S4).

Next, we interrogated whether ADH5 and ALDH2 FA detoxification might be more relevant for human cells with defects in DNA repair. To this end, we obtained Epstein–Barr virus (EBV) immortalized-lymphoblastic cells from healthy donors and from Cockayne syndrome (CSB and CSA), Fanconi anemia (FANCA), Xeroderma pigmentosa (XPC, XPA) patients and evaluated the tolerance to FA in presence of a combination of inhibitors of FA detoxification (N6022 for ADH5, and Antabuse or Disulfiram for ALDH2) (22,23) (Figure 5F, G). Our results indicate that FA detoxication confers protection independently of the cellular genetic background. However, the role of FA detoxification is strikingly relevant in cells with mutations in Fanconi anemia (*FANCA*) and in genes coding for the NER factors CSB and XPA. These data further support that FA-caused multiple DNA lesions requiring the concerted action of TC-NER, GG-NER, TCR and Fanconi anemia repair.

In sum, our results indicate that both FA-detoxification enzymes are essential to maintain proper embryonic and post-embryonic development in *C. elegans*, and that NER plays a crucial role during development and in somatic tissues in the protection of the genome from endogenously produced FA.

### The GSH-precursor NAC ameliorates FA-toxicity in *C. elegans*

**Figure 6.**
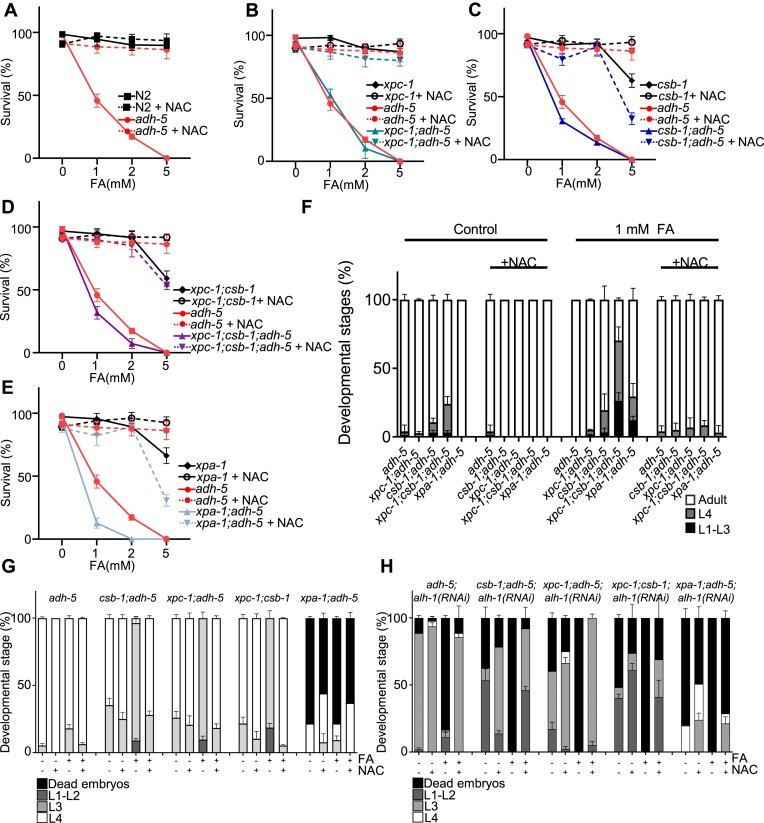
NAC suppresses formaldehyde (FA)-toxicity in NER mutants and animals lacking FA-detoxification. Panels (**A**)–(**E**) show animal survival, while (**F**) displays the developmental stages of animals treated with formaldehyde (FA) and/or N-acetyl-L-cysteine (NAC) after 72 h. (A–F) Data were produced following the same protocol as depicted in Figure 2. A full data analysis including the two-way ANOVA followed by Tukey's multiple comparisons test for (F) is available in the supplementary tables. Panels (**G**) and (**H**) were produced according to the experimental scheme used in Figure 5 and display the larval stages and dead embryos observed on the plates 48 h after treatment with FA and/or NAC. We used two-way ANOVA followed by Tukey's multiple comparisons test to determine statistical significances and a full analysis is available in the supplementary tables.

We recently showed that loss of FA clearance can lead to a disturbance of the redox homeostasis, which can be ameliorated by providing precursors of the antioxidant glutathione like the dietary supplement NAC (4). To evaluate whether ADH-5 and ALH-1-dependent FA metabolism might alter redox balance, we measured the *gst-4*::GFP reporter upon downregulation of ADH-5 and ALH-1 (Supplementary Figure S5A, S5B). Our data indicated that both ADH-5 and ALH-1 limit the activation of *gst-4*::GFP. Interestingly, the down-regulation of ALH-1 caused an striking hyperactivation of *gst-4*::GFP, while the combined downregulation of both enzymes did not show a significant further induction of *gst-4*::GFP over ALH-1 levels. The quantification of *gst-4*::GFP in the double-deficient animals might, however, be underestimated due to the high percentage of death observed in this strain. We reasoned that the NAC might directly quench FA and thus contribute to limit FA-induced oxidative stress, indicating that supplying NAC could be a therapeutic intervention to alleviate the pathologies observed in the AMeD syndrome. To test this hypothesis, we first evaluated whether NAC could protect NER-deficient animals in an *adh-5* mutant background from FA-toxicity and found that animal survival was dramatically increased (Figure 6A–E). Further, developmental delay induced by FA toxicity was completely reverted in the presence of NAC (Figure 6F). In human transformed fibroblasts from healthy donors, and from patients with mutations in *CSB, CSA, FANCA, XPA* and *XPC*, NAC was not toxic and was able to completely prevent the toxicity of FA, supporting that this dietary supplement might be suitable for alleviating FA-driven toxicity (Supplementary Figure S5C, S5D).

Then, we tested the effects of NAC in animals lacking both FA-detoxification systems ADH-5 and ALH-1. We found that both intrinsic and FA-induced embryonic lethality were largely reversed by NAC (Figure 6G and H). Specifically, the highly FA-sensitive genetic combinations *xpc-1;csb-1;adh-5;alh-1(RNAi)* and *xpa-1;adh-5;alh-1(RNAi)* displayed a robust phenotypical reversal. Together these results show that NAC is a powerful suppressor of FA-toxicity, suggesting a therapeutic intervention for patients with inherited mutations in FA-detoxifying enzymes.

## Discussion

**Figure 7.**
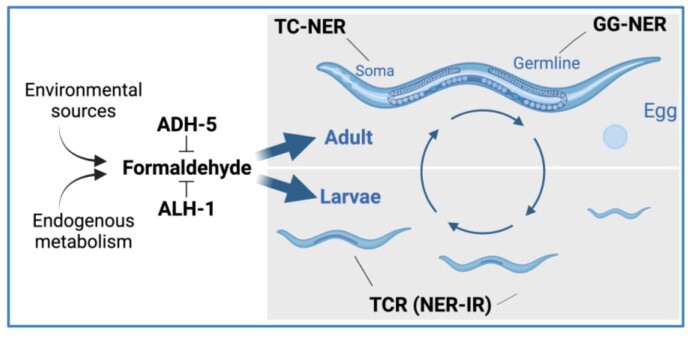
Proposed model. The scheme shows the proposed distinct functions of Global Genome Nucleotide Excision Repair (GG-NER); Transcription coupled repair (TCR) independent of NER (NER-IR); and TC-NER in somatic tissues (soma), germline, and during larvae development. ADH-5 and ALH-1 are shown to prevent systemic formaldehyde (FA) toxicity throughout *C. elegans* life cycle. Cartoons were created with BioRender.com.

FA has increasingly been recognized as an important physiological genotoxin. Here, we systematically assessed the role of a range of DNA repair mechanisms in the consequences of FA-induced damage on viability, fecundity, and aging in a metazoan organism. Our findings reveal significant contributions of multiple DNA repair pathways in resolving DNA damage induced by FA. We have found contributions of DSB-repair, BER, Fanconi anemia, SPRTN, and NER, indicating FA can cause a plethora of DNA lesions with pathogenic consequences. Specifically, we showed the distinct roles of GG-NER that is initiated by XPC-1 and TCR, which depends on CSB-1. We observe that GG-NER predominantly removes FA-induced damage in the germline, whereas TCR is primarily operating in somatic repair, as depicted in Figure 7 and summarized in Supplementary Figure S6. These results reflect the role of TCR in differentiated cell types and might be particularly relevant for the neurological pathologies caused by FA overdose as for instance demonstrated in mammalian Cockayne syndrome mouse models as well as in AMeDS patients (12,13). Interestingly, our data suggest that during larval development CSB-1 induces a NER-independent TCR, as evidenced by a minor role of the central NER factor XPA-1, while during adulthood the equal sensitivity of *xpa-1* and *csb-1* mutants suggest a canonical TC-NER function in resolving transcription-blocking FA damage. Accordingly, we determined that CSB is relevant for FA tolerance in non-dividing human cells (Figure 2I). Furthermore, we showed that CSB is involved in the resolution of FA-induced DPC (Figure 2H), which is in line with work published during the revision of this manuscript, overall supporting a NER-independent TCR of CSB in DPC resolution (24–26). It is still possible that CSB might function in the repair of other FA-induced DNA lesions such as enabling RNAPII to elongate past less obstructive lesions like oxidative base modifications (27).

Our results indicate distinct strategies for resolving FA-induced transcription blockade during development and aging. In germ cells and during the rapid cell division in embryogenesis GG-NER plays an important role as in such cell types, the entire genome needs to be cleared from FA-induced lesions. CSB-1 and XPC-1 emerge both as initial recognition factors of FA-induced DNA damage. While both operate via distinct recognition mechanisms, they share the versatility in initiating repair at distinct types of obstructive lesions. Notably, the sensitivity of the *fcd-2* (Fanconi anemia DNA repair pathway) mutant, and the heightened sensitivity of BER-deficient mutants further support the variety of genotoxic events triggered by FA treatment ranging from crosslinks and oxidative lesions.

In addition to clarifying the distinct roles of repair mechanisms in distinct tissue types, we also establish a nematode model of FA detoxification deficiencies that underlie AMeDS. By identifying the functional *ALDH2* ortholog, *alh-1*, we demonstrate that both ADH-5 and ALH-1 are required for FA detoxification, like it was reported in mice and Zebrafish (13,28). The genetic interaction observed between *adh-5, alh-1* and NER substantiates a major role of this DNA repair pathway in resolving systemic FA-induced DNA damage. Furthermore, the *adh-5;alh-1(RNAi)* model offers a platform to understand the genotoxic mechanisms driving anomalies observed in rare progeroid conditions, such as AMeDS, and possibly Cockayne syndrome or Fanconi anemia. In our settings, this AMeDS *C. elegans* model proved amenable to screening potential interventions, supported by the successful mitigation of phenotypic outcomes through the supplement NAC. The efficacy of NAC to reverse organismal pathologies of FA overload such as embryonic lethality and developmental delay suggest that this rather simple treatment could provide a valuable therapeutic intervention for patients with defects in FA detoxification enzymes (Table 1).

**Table 1.** Strain names used in this study, and listing corresponding alleles, and known mechanism-associations and the source

Strain name	Allele	Cellular mechanism	Source
N2	Wildtype		CGC
RB1801	*csb-1(ok2335)* X.	NER	CGC
TG2226	*xpc-1(tm3886)* IV.	NER	CGC
BJS21	*xpc-1(tm3886)*; *csb-1(ok2335)*	NER	Schumacher lab
FX04539	*csa-1(4539)*	NER	NBRP
FX06311	*uvs-1(tm6311)*	NER	NBRP
RB864	*xpa-1(ok698)* I.	NER	CGC
TG3867	*xpg-1(tm1670)* I.	NER/HR/BER	CGC
CB1487	*xpf-1(e1487)* II.	NER	CGC
TG1663	*ercc-1(tm1981)* I.	NER	CGC
RB877	*nth-1(ok724)* IV.	BER	CGC
RB2581	*ung-1(ok3593)* III.	BER	CGC
RB2548	*exo-3(ok3539)* I.	BER	CGC
TG1868	*slx-1(tm2644)* I.	HR/NER	CGC
NB105	*fcd-2(tm1298)* IV.	FA	CGC
NB515	*exo-1(tm1842)* III.	MMR/HR	CGC
RB964	*cku-80(ok861)* III.	NHEJ	CGC
TG1540	*gen-1(tm2940)* III.	HR	CGC
VC174	*wrn-1(gk99)* III.	HR	CGC
RB1401	*dvc-1(ok260)*	Protease	CGC
BJS886	*adh-5(sbj21)* V.	FA detox	Schumacher lab
BJS938	*xpc-1(tm3886)*;*adh-5(sbj21)*	NER/FA detox	Schumacher lab
BJS940	*xpa-1(ok698)*;*adh-5(sbj21)*	NER/FA detox	Schumacher lab
BJS885	*csb-1(ok2335)*;*adh-5(sbj21)*	NER/FA detox	Schumacher lab
BJS939	*xpc-1(tm3886)*;*csb-1(ok2335)*;*adh-5(sbj21)*	NER/FA detox	Schumacher lab

CGC, Caenorhabditis Genetics Center, University of Minnesota, MN, USA; NBRP, National BioResource Project, Japan.

## Supplementary Material

gkae519_Supplemental_Files

## Data Availability

All the data reported in this work can be found in the text and supplementary files. Reagents are available from B.S. upon request.

## Notes

Present address: Matthias Rieckher, Corporate Research and Technology (CRT), Carl Zeiss AG, Carl-Zeiss-Promenade 10, 07745 Jena, Germany.
