# Supplementary material for: Distinct DNA repair mechanisms prevent formaldehyde toxicity during development, reproduction and aging
Source: Nucleic Acids Res. 2024 Jun 19;52(14):8271–85. doi: 10.1093/nar/gkae519 (PMC11317141; doi:10.1093/nar/gkae519)
Supplement: gkae519_Supplemental_Files [file gkae519_supplemental_files.zip › Inventory list of Supplementary tablesR1.docx]

1. Supplementary tables. Statistical information for all data analysis in the figures and supplementary figures. Each sheet contains information for a panel in a figure and it is labeled accordingly.
