# Supplementary material for: Distinct DNA repair mechanisms prevent formaldehyde toxicity during development, reproduction and aging
Source: Nucleic Acids Res. 2024 Jun 19;52(14):8271–85. doi: 10.1093/nar/gkae519 (PMC11317141; doi:10.1093/nar/gkae519)
Supplement: gkae519_Supplemental_Files [file gkae519_supplemental_files.zip › Rieckher et al SuppFig_R1vf.pdf]

Supp. Fig. 1

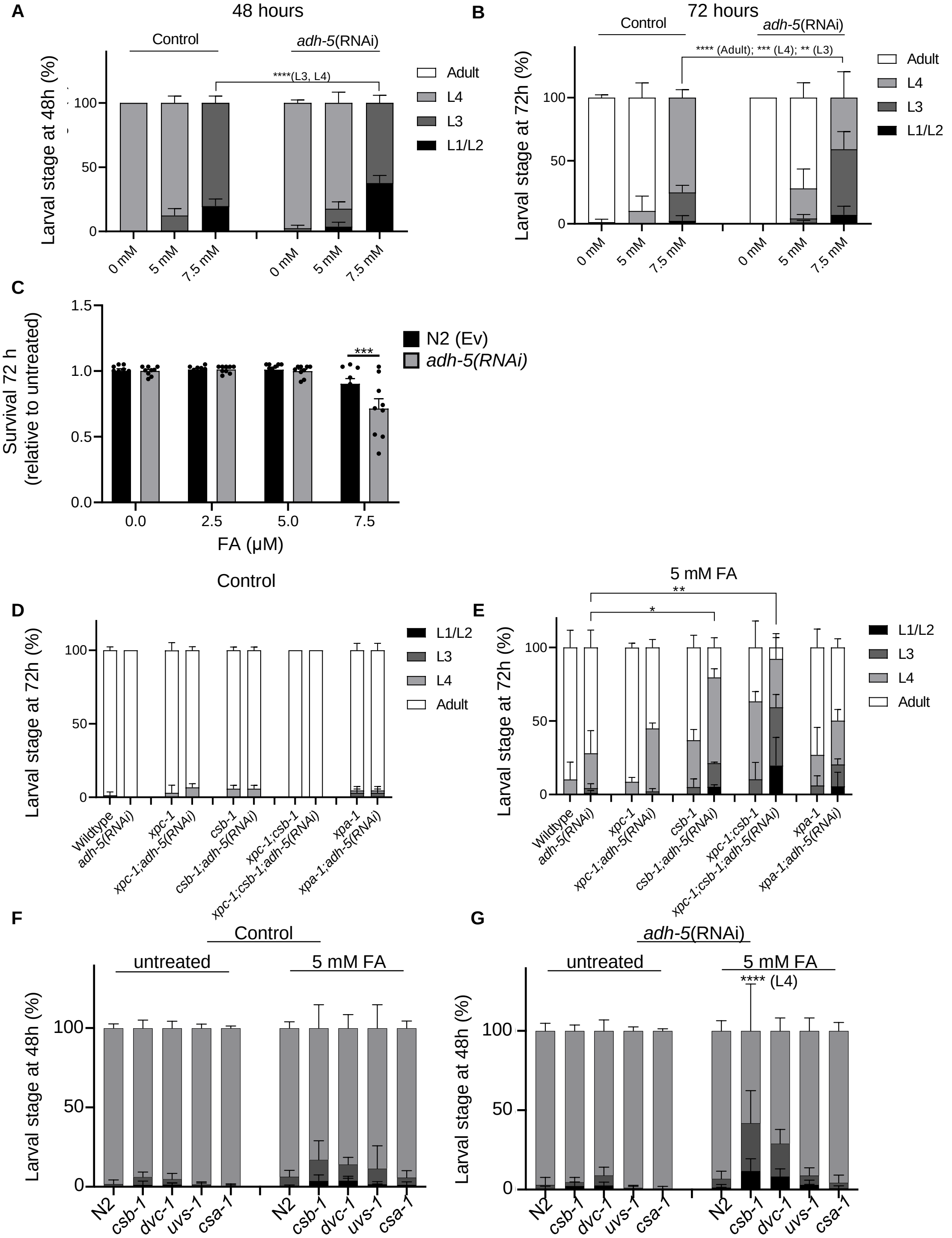

Supp. Fig. 1. Nucleotide excision repair (NER) and ADH-5 limit formaldehyde toxicity during embryo development.

A. Developmental timing of wildtype and *adh-5(*RNAi) animals upon FA after 48h on plate. Two-way ANOVA among the groups indicated (full statistic for this panel can be found as supplementary table).

B. Developmental timing of wildtype and *adh-5*(RNAi*)* animals upon FA after 72h on plate. Two-way ANOVA among the groups indicated (full statistic for this panel can be found as supplementary table).

C Survival at 72h depicted as ratio of the surviving animals in the untreated condition for each strain (n=9, mean +/- SEM, Two-way ANOVA ****p* = 0.0004)).

D. Developmental timing of NER mutants grown on *adh-5*(RNAi) animals after 72h on plate.

E. Developmental timing of NER mutants grown on *adh-5*(RNAi) animals upon FA after 72h on plate.

F. Developmental stage reached by larvae of NER mutants recorded a 48h in presence or absence of 5 mM FA.

G. Developmental stage reached by NER mutants in *adh-5*(RNAi) recorded a 48h in presence or absence of 5 mM FA. Two-way ANOVA ****p* < 0.0001 between *csb-1* or *dvc-1* and N2.

Supp. Fig. 2

A

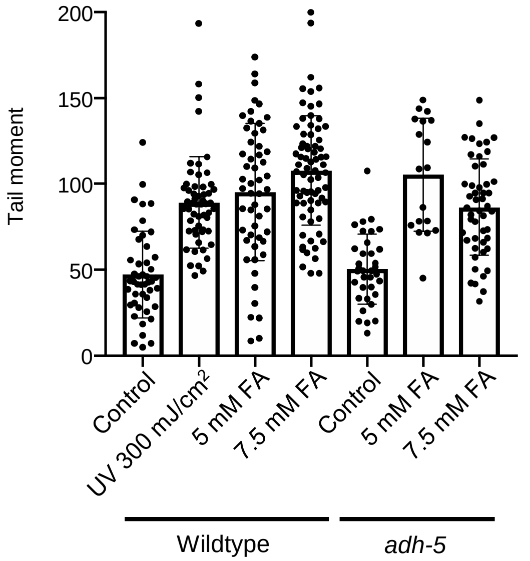

B

Tukey's multiple comparisons test	Summary	Adjusted P Value
WT vs. WT 300mJ UV/cm^2	****	<0.0001
WT vs. WT 7.5 mM FA	****	<0.0001
WT vs. *adh-5*	ns	0.9994
WT vs. *adh-5* 7.5 mM FA	****	<0.0001
WT vs. WT 5 mM FA	****	<0.0001
WT vs. *adh-5* 5 mM FA	****	<0.0001
WT300mJ UV/cm^2 vs. WT 7.5 mM FA	*	0.0112
WT300mJ UV/cm^2 vs. *adh-5*	****	<0.0001
WT300mJ UV/cm^2 vs. *adh-5* 7.5 mM FA	ns	0.9993
WT300mJ UV/cm^2 vs. WT 5 mM FA	ns	0.9396
WT300mJ UV/cm^2 vs. *adh-5* 5 mM FA	ns	0.4264
WT 7.5 mM FA vs. *adh-5*	****	<0.0001
WT 7.5 mM FA vs. *adh-5* 7.5 mM FA	**	0.0030
WT 7.5 mM FA vs. WT 5 mM FA	ns	0.2526
WT 7.5 mM FA vs. *adh-5* 5mM FA	ns	>0.9999
adh-5 vs. *adh-5* 7.5 mM FA	****	<0.0001
adh-5 vs. WT 5 mM FA	****	<0.0001
adh-5 vs. *adh-5* 5mM FA	****	<0.0001
adh-5 7.5 mM FA vs. WT 5 mM FA	ns	0.7546
adh-5 7.5 mM FA vs. *adh-5* 5mM FA	ns	0.2554
WT 5 mM FA vs. *adh-5* 5mM FA	ns	0.8825

C

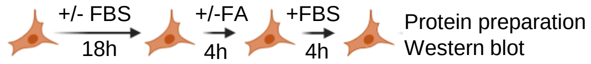

D

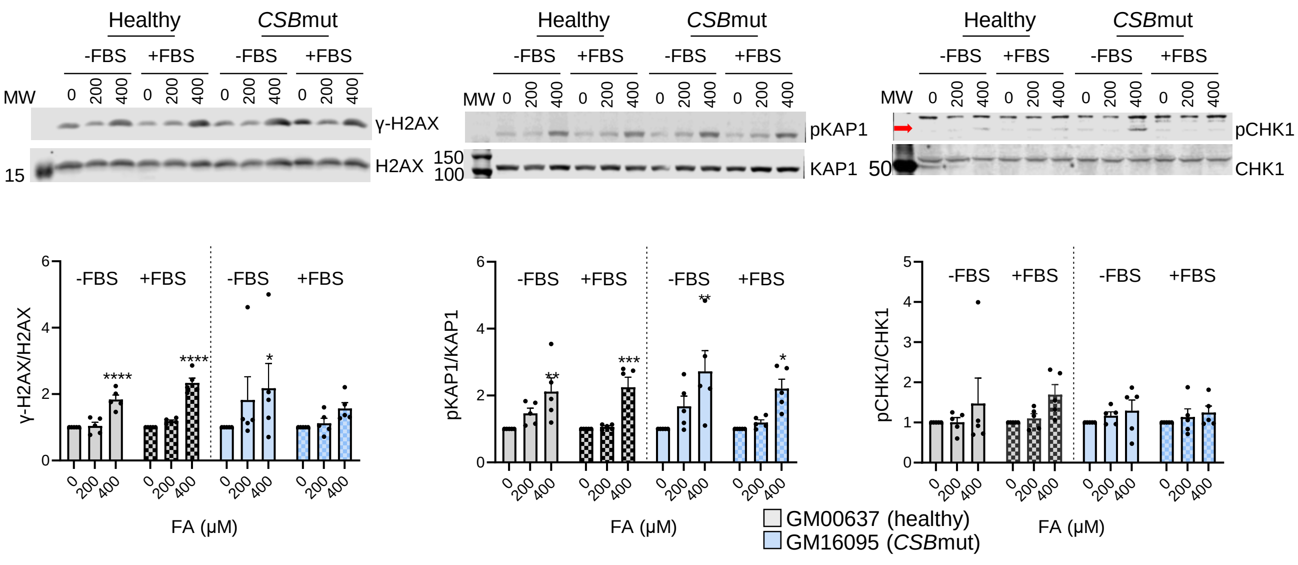

Supp. Fig. 2. Formaldehyde (FA) induces double-strand breaks and DNA damage markers

A. Comet assay on day-1-adult animals 24 h post-exposure to the indicated concentrations of FA or UVB.

B. Statisticical analysis for the data shown in A using the one-way ANOVA followed by the Tukey's multiple comparisons test.

C. Scheme showing the experimental protocol for syncronization of cells in G0/G1 and FA treatment for immunoblots.

D. Representative immunoblots of the DNA damage markers γ-H2AX, pKAP1, and pCHK1 determined 4 hours after treatment in synchronized non-dividing and dividing cells. In the bottom plots the quantification is shown (n=5, SEM, Two-way ANOVA between the FA and the untreated condition for each set corrected with a Sidak´s test).

Supp. Fig. 3

A

```
NP_498081.2 ALH-1 -------MLRSALRATVQARNASGVPPGLSNMKPQYTGIFINNEFVPAKSGKTFETINPA
NP_503467.2 ALH-2 ---MDSSLLRSAVRASVQACNGSGLPPGLADFKPKYTSLFINNEFVDAKSGKTFEFVNPA
hALDH2            MLRAAARFGPRLGRRLLSAAATQAVPAPNQQPEVFCNQIFINNEWHDAVSRKTFPTVNPS

NP_498081.2 ALH-1 NGKVLAQVAEGDKTDVNIAVKAAQNAFRIGSEWRRMDASQRGVLLNRLADLMERDRVILA
NP_503467.2 ALH-2 NGKLLAKVAEGNRDDVDIAVEAAKKAFKIGSEWRRMDASHRGVLLNRLADLMERDRVILA
hALDH2            TGEVICQVAEGDKEDVDKAVKAARAAFQLGSPWRRMDASHRGRLLNRLADLIERDRTYLA

NP_498081.2 ALH-1 SLESLDNGKPYAVAYNADLPLSIKTLRYYAGWADKNHGKTIPIEGDYFTYTRHEPVGVCG
NP_503467.2 ALH-2 SLESLDNGKPYKEAYNIDLPISIKTFRYYAGYADKNHGKTIPVGGDYFTYTRHEPVGVCG
hALDH2            ALETLDNGKPYVISYLVDLDMVLKCLRYYAGWADKYHGKTIPIDGDFFSYTRHEPVGVCG

NP_498081.2 ALH-1 QIIPWNFPLLMQAWKLGPALAMGNTVVMKVAEQTPLSALHVAALTKEAGFPDGVVNIIPG
NP_503467.2 ALH-2 QIIPWNFPLLMQAWKLAPALAMGNTVVMKVAVKTPLSALHVASLIKEAQFPEGVVNIIPG
hALDH2            QIIPWNFPLLMQAWKLGPALATGNVVVMKVAEQTPLTALYVANLIKEAGFPPGVVNIVPG

NP_498081.2 ALH-1 YGHTAGQAISSHMDVDKVAFTGSTEVGRLVMKAAAESNVKKVTLELGGKSPNIIFADADL
NP_503467.2 ALH-2 RGTDAGEAIASHMDVDKVAFTGSTEVGKTIMKAAAESNVKKVTLELGGKSPNIVFADADL
hALDH2            FGPTAGAAIASHEDVDKVAFTGSTEIGRVIQVAAGSSNLKRVTLELGGKSPNIIMSDADM

NP_498081.2 ALH-1 NDSVHQANHGLFFNQGQCCCAGSRTFVEGKIYDDFVARSKELAEKAVIGDPFDLKTTQGP
NP_503467.2 ALH-2 EEAVRQSHHALFFNQGQCCSAGSRTFVEGKIYDEFVAKAKELVEKTVIGDPFDENTTQGP
hALDH2            DWAVEQAHFALFFNQGQCCCAGSRTFVQEDIYDEFVERSVARAKSRVVGNPFDSKTEQGP

NP_498081.2 ALH-1 QVDGKQVETILKYIAAGKKDGAQLVTGGAKHGDQGHFVKPTIFANVKDQMTIAQEEIFGP
NP_503467.2 ALH-2 QIDESQVETIMKYIESGKKEGAQLVTGGVKHGDQGYFVKPTIFANVNDQMKIAQEEIFGP
hALDH2            QVDETQFKKILGYINTGKQEGAKLLCGGGIAADRGYFIQPTVFGDVQDGMTIAKEEIFGP

NP_498081.2 ALH-1 VMTIIRFDTMEELVEKANNTIYGLAAGVMTKDIDKALHIANATRAGSVWVNCYDVFDAAA
NP_503467.2 ALH-2 VMIVIRFDSMEELIEKANNTIYGLAAGVVTNDLNKALQVANTIRAGSVWVNCYDVFDPAA
hALDH2            VMQILKFKTIEEVVGRANNSTYGLAAAVFTKDLDKANYLSQALQAGTVWVNCYDVFGAQS

NP_498081.2 ALH-1 PFGGFKQSGIGRELGEYGLEAYTEVKTVTIKVPQKNS
NP_503467.2 ALH-2 PFGGFKQSGIGRELGEYGLAAYTEVKTVTIKVPQKNS
hALDH2            PFGGYKMSGSGRELGEYGLQAYTEVKTVTVKVPQKNS
```

B

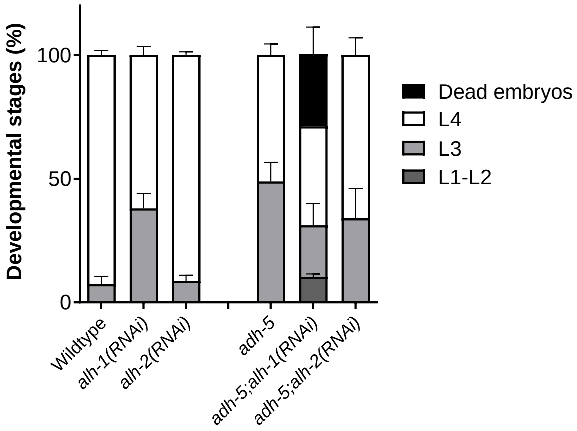

Supp. Fig. 3. ALH-1 is the ortholog of human ALDH2.
A. Alignment of human ALDH2, ALH-1, and ALH-2. The conserved NAD+ binding site is highlighted in yellow. The mutation of the active Cys to Ser found in ALH-2 is depicted in red.
B. ALH-1, but not ALH-2, interacts with ADH-5, impairing *C. elegans* development (related to Fig. 5).

Supp. Fig. 4

A

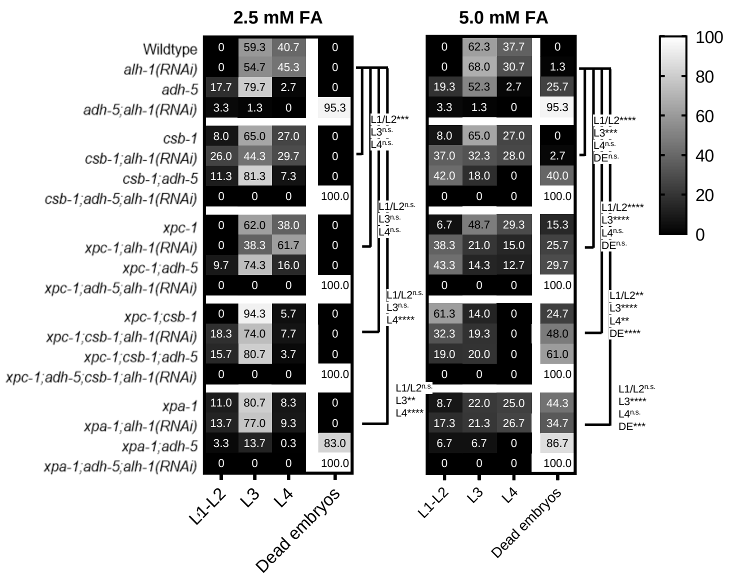

Supp. Fig. 4. Formaldehyde (FA) induces embryonic lethality and development delay in aldehyde detoxification deficient backgrounds.

A. Heatmap combining data of development and embryonic lethality of animals exposed to *alh-1(RNAi)*. The displayed values are the mean percentages of events counted on the plates (stages and dead embryos) 48 h after FA-exposure (2.5 mM and 5.0 mM) for three technical replicates. The significance was determined via the two-way ANOVA, followed by the Tukey's multiple comparison test, for which counts *$p < 0.05$, **$p < 0.01$, ***$p < 0.001$, ****$p < 0.0001$. A full statistical analysis between all the groups is available in Supplementary Table 7.

Supp. Fig. 5

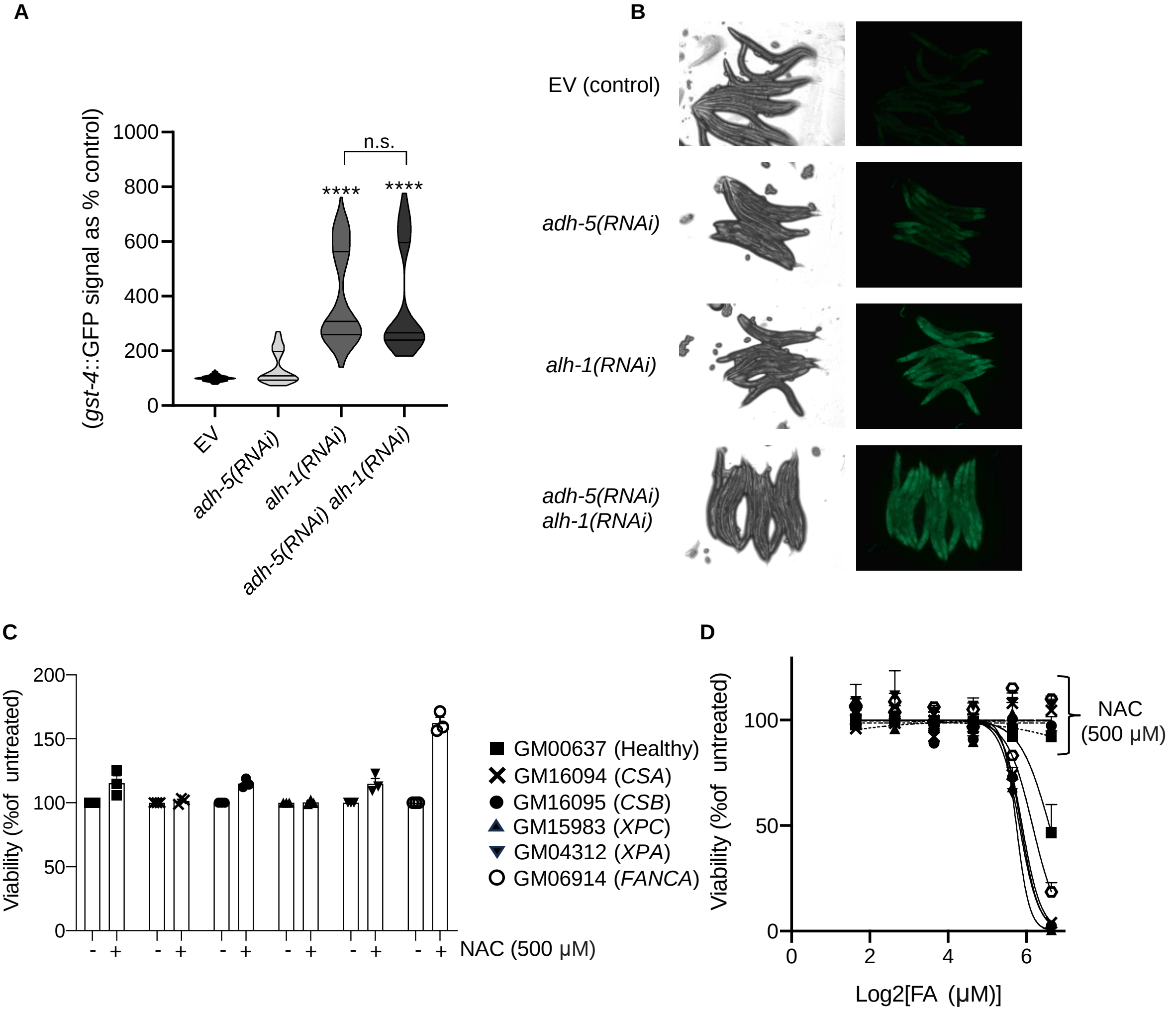

Supp. Fig. 5. N-acetyl-L-cysteine (NAC) limits formaldehyde (FA) toxicity in human fibroblasts derived from patients
A. Quantification of pixel intensity from images taken in animals carrying the *gst-4::GFP* reporter and plotted as a percentage of the EV control. Each value represents the intensity of one animal (EV (control) n=80; *adh-5(RNAi)* n=80; *alh-1(RNAi)* n=92*; adh-5(RNAi) alh-1(RNAi)* n=82). One-way ANOVA corrected with a Tukey test between each condition and the EV (*****p* <0.0001).
B. Representative images used for quantifications in C.
C. NAC is not toxic to SV40 transformed human fibroblasts with mutations in genes coding for the DNA repair factors CSB, CSA, XPA, XPC, FANCA nor for fibroblasts from an apparently healthy donor.
D. NAC at 500 µM can prevent the toxicity of FA independently of the genetic background of the fibroblasts.

Supp. Fig. 6

Strain	Formaldehyde stress			
	Survival	Development	Fecundity	Lifespan
csb-1	+	+	+	-
xpc-1	-	-	+	NT
csb-1:xpc-1	+	+	+	NT
xpa-1	-	+	+	+
xpg-1	NT	+	NT	NT
xpf-1	NT	-	NT	NT
ercc-1	NT	+	NT	NT
nth-1	NT	-	NT	NT
ung-1	NT	-	NT	NT
exo-3	NT	-	NT	NT
dvc-1	+	+	NT	NT
csa-1	-	-	NT	NT
uvs-1	-	-	NT	NT
adh-5	+	-	+	+
xpc-1;adh-5	+	-	+	+
csb-1;adh-5	+	+	+	+
xpc-1;csb-1;adh-5	+	+	+	NT
xpa-1;adh-5	+	+	+	+

Survival: data from Fig. 2

Fecundity: Eggs laid/3 h from Fig. 3

Development: Data from Fig. 2, and Supp. Fig. 1

Lifespan: data from Fig. 4

Supp. Fig. 6 Table showing the summary of phenotypes for strains tested in Fig. 2, Fig.3, and Fig. 4 under formaldehyde stress.
The phenotypes are presented relative to the N2 control in each assay and recorded as "+" is there is an effect between the strain tested and the N2 control in conditions of formaldehyde (FA) stress. "-" indicates no phenotype, while NT indicate not tested.
